# Burden of kidney disease on the discrepancy between reasons for hospital admission and death: An observational cohort study

**DOI:** 10.1371/journal.pone.0258846

**Published:** 2021-11-03

**Authors:** Shintaro Mandai, Fumiaki Ando, Takayasu Mori, Koichiro Susa, Soichiro Iimori, Shotaro Naito, Eisei Sohara, Shinichi Uchida, Kiyohide Fushimi, Tatemitsu Rai

**Affiliations:** 1 Department of Nephrology, Graduate School of Medical and Dental Sciences, Tokyo Medical and Dental University, Bunkyo, Tokyo, Japan; 2 Department of Health Policy and Informatics, Graduate School of Medical and Dental Sciences, Tokyo Medical and Dental University, Bunkyo, Tokyo, Japan; Shizuoka General Hospital: Shizuoka Kenritsu Sogo Byoin, JAPAN

## Abstract

**Background:**

Physicians have long noted a substantial discrepancy between the reasons for hospital admission and ultimate causes of death, particularly among older adults or patients with complex underlying diseases. However, objective data on this phenomenon are lacking. We aimed to examine the risk of in-hospital death caused by a reason other than the original reason for hospitalization and its association with underlying kidney disease in a nationwide inpatient database.

**Methods:**

In this retrospective cohort study, we studied 639,556 Japanese adults who died in the hospital from 2012 to 2015, using data from Japan’s Diagnosis Procedure Combination database. We analyzed the discrepancy rate between reasons for hospital admission and death and associated factors using the International Classification of Diseases, 10th Revision (ICD-10) diagnostic codes and seven related categories.

**Results:**

Among non-chronic kidney disease (CKD) (590,551), CKD (24,708), and end-stage kidney disease (ESKD) (24,297) patients, the median age was 77 years (interquartile range [IQR]: 67–84 years), 83 years (IQR: 75–88), and 75 years (IQR: 67–81), and 25.7%, 30.3%, and 41.6% died from a reason other than the original reason for hospitalization, respectively. Multivariate logistic regression analyses determined CKD/ESKD as the predominant risk factor for this discrepancy, rather than older age, male sex, obesity, and other comorbidities. Sankey diagrams that presented diagnostic changes from hospital admission to death revealed multiple wider segments connecting to different disease classifications, particularly to congestive and septic death in CKD and ESKD patients, respectively. Death owing to another disease classification led to an increase in the median length of hospital stay by 5–7 days and to a 1.3-–1.4-fold increase in medical costs across the populations.

**Conclusions:**

A substantial proportion of patients with CKD and ESKD died during hospitalization for a reason other than their original reason for admission, leading to increased length of hospital stay and cost.

## Introduction

Clinical experience indicates that in a substantial proportion of patients, clinical status dynamically changes and death is caused by reasons other than the original diagnosis at hospital admission. This discrepancy is particularly evident among older adults or patients with underlying complex diseases including malignancy disease, cardiovascular disease, or chronic kidney disease (CKD). These populations are more prone to having multiple comorbidities at hospital admission or developing unexpected complications afterward, ultimately leading to unfavorable outcomes and increased healthcare expenditures [1–3]. However, a comprehensive understanding of these clinical cases is lacking as previous investigations have focused on specific clinical situations such as deaths after pneumonia, heart failure, trauma, or fracture [4–8].

CKD is a major chronic noncommunicable disease (NCD) that globally affects 9%–15% of patients [9–11]. It can progress to end-stage kidney disease (ESKD) predominantly through diabetes mellitus and hypertension. Although CKD is a partially treatable and preventable NCD given current advancements in diagnoses, treatment, and healthcare, the rates of need for renal replacement therapy and CKD-related mortality are rising [11]. Worldwide, an estimated 700 million people have CKD, and its disability-adjusted life years reached 35.8 million in 2017 [11]. A main reason behind these issues could be kidney and distant organ crosstalk leading to disorders of multiple organs (e.g., cardiovascular disease, stroke, dementia, bone fragility, or sarcopenia) [1,11–15].

Because CKD is a systemic complex disease, we hypothesize that the complexity of patients’ problems, difficulties in prompt diagnoses, or newly occurring comorbidities cause greater dynamics in patient status and discrepancy between diagnosis on hospital admission and the ultimate cause of in-hospital death, leading also to higher length of stay and care expenditures for CKD or ESKD patients. Accordingly, we measure the rates of discrepancy between reasons for hospital admission and death and associated risk factors. The aim is to visualize the transition of disease categories in patients with pre-dialysis CKD and ESKD who died during hospitalization.

## Materials and methods

### Data source

Data were taken from the Diagnosis Procedure Combination (DPC) national inpatient database in Japan for the years 2012–2015 [14,16,17]. The DPC uses a case-mix classification system to ensure the transparency of hospital performance, which is linked with a payment system. Over 1,000 hospitals in Japan, including all 82 teaching hospitals, contribute to it, covering approximately 50% of all hospital admissions in the country. The DPC includes administrative claims and discharge abstract data such as a unique hospital identifier; patient’s age on admission and sex; diagnoses and comorbidities at hospital admission, coded according to the International Classification of Diseases and Related Health Problems, 10th Revision (ICD-10) [18]; Charlson Comorbidity Index (CCI) [19], updated and modified for use with ESKD patients [14,20]; and discharge status. Inpatient physicians are in charge of the accuracy of the recorded information.

This retrospective cohort study was performed after the ethics committee of the Tokyo Medical and Dental University approved the research (No. M2000-788) and waived the need for informed consent to ensure data anonymity. The study was performed in accordance with the ethical principles of the Declaration of Helsinki.

### Participants

There were 29,358,759 eligible hospital admissions in the chosen timeframe. The criteria for participants were as follows: age ≥18 years, hospitalization ≥24 h, and death during hospitalization with complete information on the reason for admission and mode of death. CKD patients were identified when the ICD-10 codes of the primary diagnoses or comorbidities included either of N180, N188, or N189. The ICD-10 codes N181, N182, N183, N184, and N185 that correspond to each CKD grade has yet to be recorded in the database. Patients with ESKD on maintenance hemodialysis or peritoneal dialysis were identified based on the code of patient care procedures: chronic maintenance hemodialysis with <4 hours per session, ≥4 hours and <5 hours per session, ≥5 hours per session, chronic maintenance hemodiafiltration, or continuous peritoneal dialysis [17]. Patients with incomplete information regarding patient characteristics at admission and those with a body mass index (BMI) under 15 kg/m^2^ or over 50 kg/m^2^ were excluded.

Other patient-level data included age, sex, and fiscal year of admission. The hospital-level variables were hospital volume (HV), which represents the mean daily number of hospitalized patients with and without ESKD, and dialysis case volume (DCV), which is the mean annual number of hospitalized patients who received maintenance dialysis [17].

### Reason for hospital admission and cause of death

The database normally records the reason for hospital admission based on ICD-10 diagnosis codes but does not fully specify the direct cause of death. To categorize the disease diagnoses into a clinically meaningful classification, we sorted them into seven categories based on the Healthcare Cost and Utilization Project (HCUP) mapping of ICD-10 diagnosis codes (S1 Table): vascular event, congestion, sepsis, cancer, falls/fracture/trauma, vascular access, and other [21,22]. The medical costs needed for patient care during hospitalization were documented using the exchange rate from Japanese yen to United States dollars (USD) as of September 9, 2020.

### Statistical analysis

Continuous or categorical variables were reported as medians and interquartiles or numbers with percentages. Logistic regression analyses were performed to examine the factors associated with the risk of in-hospital death from a reason other than that reported for initial hospitalization. The logistic regression models were adjusted for age, sex, BMI, CCI, admission type, and admission year. Sankey diagrams were generated using Google Chart (https://developers.google.com/chart) to visualize how primary diagnostic classifications were altered from admission until death during patients’ hospitalization. All statistical analyses were conducted with Stata (version 15.0; Stata Corp., College Station, TX, USA). Finally, *P*-values <0.05 were considered statistically significant.

## Results

### Characteristics of Japanese adults who died in the hospital

In total, 809,873 adults who were admitted to Japanese hospitals from 2012 to 2015 died of clarified diagnoses during hospitalization. Among them, 639,556 patients were included in the analysis (S1 Fig). Table 1 shows the patients’ characteristics and reasons for hospital admission. There were 590,551 non-CKD adults, 24,708 CKD adults, and 24,297 ESKD adults. The median age was 77 years (interquartile range [IQR]: 68–84 years), 83 years (IQR: 75–88), and 75 years (IQR: 67–81), and 41%, 39%, and 32% of the group members were female, respectively. The non-CKD group had a greater prevalence of lean patients. Moreover, ESKD and CKD adults were more likely to be diabetic. The prevalence of underlying cardiovascular disease and emergent admission was higher in the CKD group.

**Table 1:** Characteristics of Japanese adults who died in the hospital from 2012 to 2015.

Characteristic	Non-CKD (*N* = 590,551)	CKD (*N* = 24,708)	ESKD (*N* = 24,297)
Patient-level			
Age (year)	77 (67–84)	83 (75–88)	75 (67–81)
≤ 64, N (%)	109,452 (19)	1,979 (8)	4,797 (20)
65–74, N (%)	144,230 (24)	3,972 (16)	7,027 (29)
≥ 75, N (%)	336,869 (57)	18,757 (76)	12,473 (51)
Female, N (%)	239,261 (41)	9,525 (39)	7,780 (32)
BMI (kg per m^2^)	20 (18–23)	21 (18–23)	21 (18–23)
≤ 18, N (%)	179,640 (30)	6,461 (26)	6,144 (25)
19–24, N (%)	322,203 (55)	13,991 (57)	13,799 (57)
25–29, N (%)	74,017 (13)	3,485 (14)	3,487 (14)
≥ 30, N (%)	14,691 (2)	771 (3)	867 (4)
CCI			
Cardiovascular disease, N (%)	108,683 (18)	8,876 (36)	7,829 (32)
Diabetes mellitus, N (%)	87,598 (15)	5,419 (22)	6,669 (27)
CCI score			
0, N (%)	243,582 (41)	11,003 (45)	11,190 (46)
1–2, N (%)	146,146 (25)	9,209 (37)	9,049 (37)
≥ 3, N (%)	200,823 (34)	4,496 (18)	4,058 (17)
Emergent admission, N (%)	478,507 (81)	21,932 (89)	18,676 (77)
Reason for admission			
ICD-10 diagnosis codes			
Infectious and parasitic diseases (A00-B99), N (%)	13740 (2)	933 (4)	1478 (6)
Neoplasm and hematopoietic disorders (C00-D89), N (%)	278608 (47)	4321 (17)	4223 (17)
Endocrine, nutritional, and metabolic diseases (E00-E90), N (%)	13761 (2)	837 (3)	503 (2)
Diseases of the nervous system and mental disorders (F00-G99), N (%)	6424 (1)	159 (1)	207 (1)
Diseases of the eyes and ears (H00-H95), N (%)	266 (0.05)	7 (0.03)	23 (0.1)
Diseases of the circulatory system (I00-I99), N (%)	92886 (16)	7654 (31)	6227 (26)
Diseases of the respiratory system (J00-J99), N (%)	96521 (16)	3872 (16)	2836 (12)
Diseases of the digestive system (K00-K93), N (%)	40372 (7)	1548 (6)	2210 (9)
Diseases of the musculoskeletal system, skin, and soft tissue (L00-M99), N (%)	5075 (1)	333 (1)	874 (4)
Diseases of the genitourinary system (N00-N99), N (%)	8162 (1)	3516 (14)	3484 (14)
Injury and poisoning (S00-T81, T88-T98, V01-Y98), N (%)	14684 (2)	672 (3)	953 (4)
Vascular access (T82-T87), N (%)	516 (0.1)	52 (0.2)	456 (2)
Others, N (%)	19536 (3)	804 (3)	823 (3)
Clinical disease classification			
Vascular event, N (%)	65193 (11)	3256 (13)	4037 (17)
Congestion, N (%)	23036 (4)	4074 (16)	1693 (7)
Sepsis, N (%)	52671 (9)	2899 (12)	3057 (12)
Cancer, N (%)	272133 (46)	4040 (16)	3851 (16)
Falls/fracture/trauma, N (%)	7770 (1)	369 (1)	524 (2)
Vascular access, N (%)	516 (0.1)	52 (0.2)	456 (2)
Other, N (%)	169232 (29)	10018 (41)	10679 (44)
Year			
2012, N (%)	149,453 (25)	6,081 (25)	6,598 (27)
2013, N (%)	123,256 (21)	5,016 (20)	4,851 (20)
2014, N (%)	171,555 (29)	7,159 (29)	7,221 (30)
2015, N (%)	146,287 (25)	6,452 (26)	5,627 (23)
Hospital-level			
Hospital volume (mean admissions per day)	322 (201–479)	296 (182–453)	369 (223–550)
Annual dialysis case volume (mean dialysis admissions per year)	336 (124–621)	336 (134–625)	516 (290–810)

The data are numbers (percentiles) or medians (interquartile ranges) on hospital admission. BMI, body mass index; CKD, chronic kidney disease; ESKD, end-stage kidney disease; ICD-10, International Classification of Diseases, 10th Revision.

### Reason for hospital admission

There was discernible heterogeneity in the reason for hospital admission across the three groups, particularly between the non-CKD and CKD/ESKD groups (Table 1). In the non-CKD group, the leading causes of admission were neoplasm and hematopoietic disorders, followed by diseases of the circulatory and respiratory systems. Conversely, in the CKD and ESKD groups, the most frequent reason for admission was diseases of the circulatory system, followed by neoplasm and hematopoietic disorders and diseases of the respiratory system. Admission for diseases of the eyes, ears, musculoskeletal system, skin, and soft tissue resulted in death during hospitalization for a considerably small proportion of the entire study group. A small prevalence of admission with diseases of the genitourinary system was seen in the non-CKD group, and 14% of CKD/ESKD adults died after hospitalization because of disorders of the genitourinary system.

To classify the diagnoses with ICD-10 codes into more meaningful categories, we sorted them into seven HCUP groups [21,22]. This clinical classification showed that nearly half of patients with non-CKD died after being hospitalized for cancer (Table 1). Vascular events were among the major reasons for hospital admission in all the groups; this prevalence was particularly high in ESKD adults. Congestion was the primary reason for hospital admission leading to death, with a prevalence rate similar to cancer in the CKD population. Admission for sepsis was more likely to lead to in-hospital death for CKD and ESKD patients. A small proportion of patients with ESKD died after vascular access-associated admission. A higher proportion of patients with ESKD were admitted to hospitals with a higher HV and DCV [17].

### Discrepancy between diagnoses on admission and in-hospital death and the associated factors

**Fig 1:**
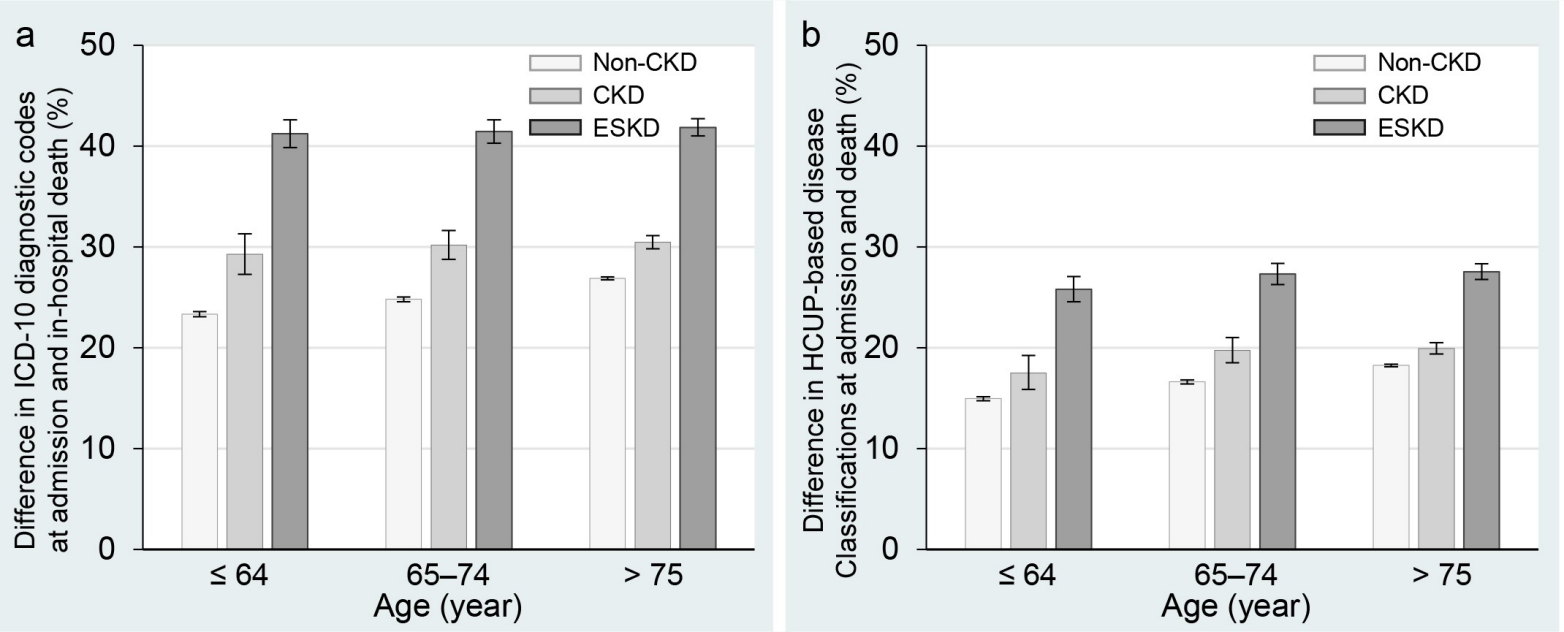
Rate of discrepancy between primary reasons for hospital admission and death among Japanese adults. **a)** Rate of discrepancy between primary ICD-10 diagnostic code assigned at the time of hospital admission and death among Japanese adults with non-CKD, CKD, and ESKD. **b)** Rate of death owing to different HCUP-based disease classification following hospital admission among Japanese adults. Each bar graph represents a mean, and the solid lines represent the corresponding 95% confidence interval. CKD, chronic kidney disease; ESKD, end-stage kidney disease; HCUP, Healthcare Cost and Utilization Project; ICD-10, International Classification of Diseases, 10th Revision.

The rate of discrepancy between ICD-10 codes on hospital admission and in-hospital death was noticeably high: 41.6% in ESKD patients, 30.3% in CKD patients, and 25.7% in non-CKD patients (Fig 1A). Moreover, in the non-CKD population, it increased with patients’ age. For CKD or ESKD patients, however, the non-elderly showed a high discrepancy rate similar to the older people. When analyzed using the clinical disease classification, the discrepancy between the reason for admission and direct cause of death was almost twice as high in ESKD patients compared to non-CKD patients (Fig 1B). We also analyzed the mismatch between the direct cause of death and the secondary comorbidities overlapping with the primary diagnosis on admission. As a result, there were only 9,157 deaths (5.4%) due to any of the secondary diagnoses at admission. Thus, the discrepancy rates between the multiple diagnoses including comorbidities at admission and the cause of death were very similar to those in the original analysis (Fig 1), as shown in S2 Fig.

**Fig 2:**
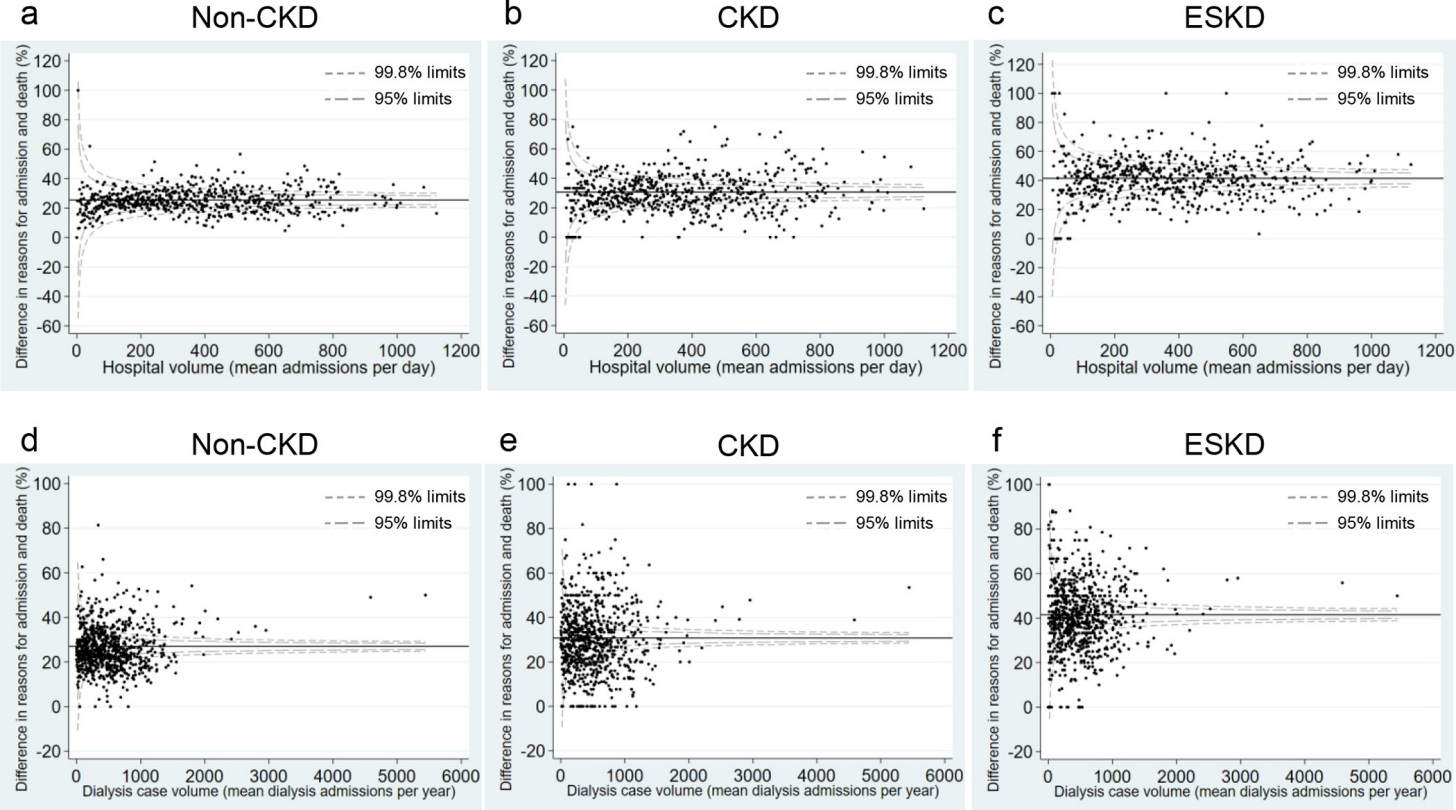
Funnel plots for hospital volume and discrepancy between reasons for hospital admission and in-hospital death. **a–c)** Hospital volume is defined as the mean number of daily hospitalized patients. **d–f)** Dialysis case volume is defined as the mean number of annually hospitalized patients on maintenance dialysis. Each hospital is represented by a point on the Funnel plots showing the rate of difference in the reason for hospital admission and death, among the total admissions leading to death in each hospital. The control limits represent the expected range of discrepancy rate between reasons for hospital admission and death based on the Poisson distribution. CKD, chronic kidney disease; ESKD, end-stage kidney disease.

To investigate whether HV factors affected the discrepancy between the reasons for hospital admission and death across the groups, we illustrated the Funnel plots [23] of the observed and expected range of the discrepancy rate of ICD-10 codes based on the Poisson distribution for HV representing mean admissions per day and DCV [17] representing mean annual dialysis admissions per year (Fig 2). The rate of discrepancy was found to be independent of HV factors across the groups and remained high for CKD and ESKD patients even when these patients were treated in hospitals with a large HV or DCV.

To investigate the factors associated with a high discrepancy between the reasons for hospital admission and death, we performed multivariate logistic regression analyses. CKD/ESKD, higher age, male sex, obesity, and other comorbidities were associated with a greater discrepancy between the reason for hospital admission and cause of death (Table 2). Emergent admission was associated with the discrepancy. The logistic regressions using clinical disease classification also revealed increased odds of the discrepancy in patients who had CKD/ESKD, had a higher age, were male, were overweight, and had other comorbidities (S2 Table).

**Table 2:** Risk factors for a discrepancy between reasons for hospital admission and death in Japanese adults.

Variable	OR (95% CI)	*P* value
Kidney disease		
Non-CKD	Reference	
CKD	1.221 (1.187–1.256)	<0.001
ESKD	2.223 (2.165–2.283)	<0.001
Age (year)		
≤64	Reference	
65–74	1.084 (1.065–1.103)	<0.001
≥75	1.178 (1.160–1.197)	<0.001
Sex		
Female vs. male	0.968 (0.957–0.980)	<0.001
BMI (kg per m^2^)		
≤18	1.006 (0.993–1.019)	0.4
19–24	Reference	
25–29	1.051 (1.033–1.070)	<0.001
≥30	1.166 (1.125–1.207)	<0.001
CCI score	1.042 (1.040–1.045)	<0.001
Admission type		
Emergent vs. elective	1.904 (1.873–1.935)	<0.001
Year		
2012	Reference	
2013	0.952 (0.937–0.968)	<0.001
2014	0.912 (0.898–0.926)	<0.001
2015	0.837 (0.824–0.851)	<0.001

Multivariate logistic regression models were adjusted for age, sex, BMI, Charlson Comorbidity Index, and admission type and year. BMI, body mass index; CI, confidence interval; CKD, chronic kidney disease; ESKD, end-stage kidney disease; OR, odds ratio.

### Diagnostic changes from hospital admission to eventual death during hospitalization

**Fig 3:**
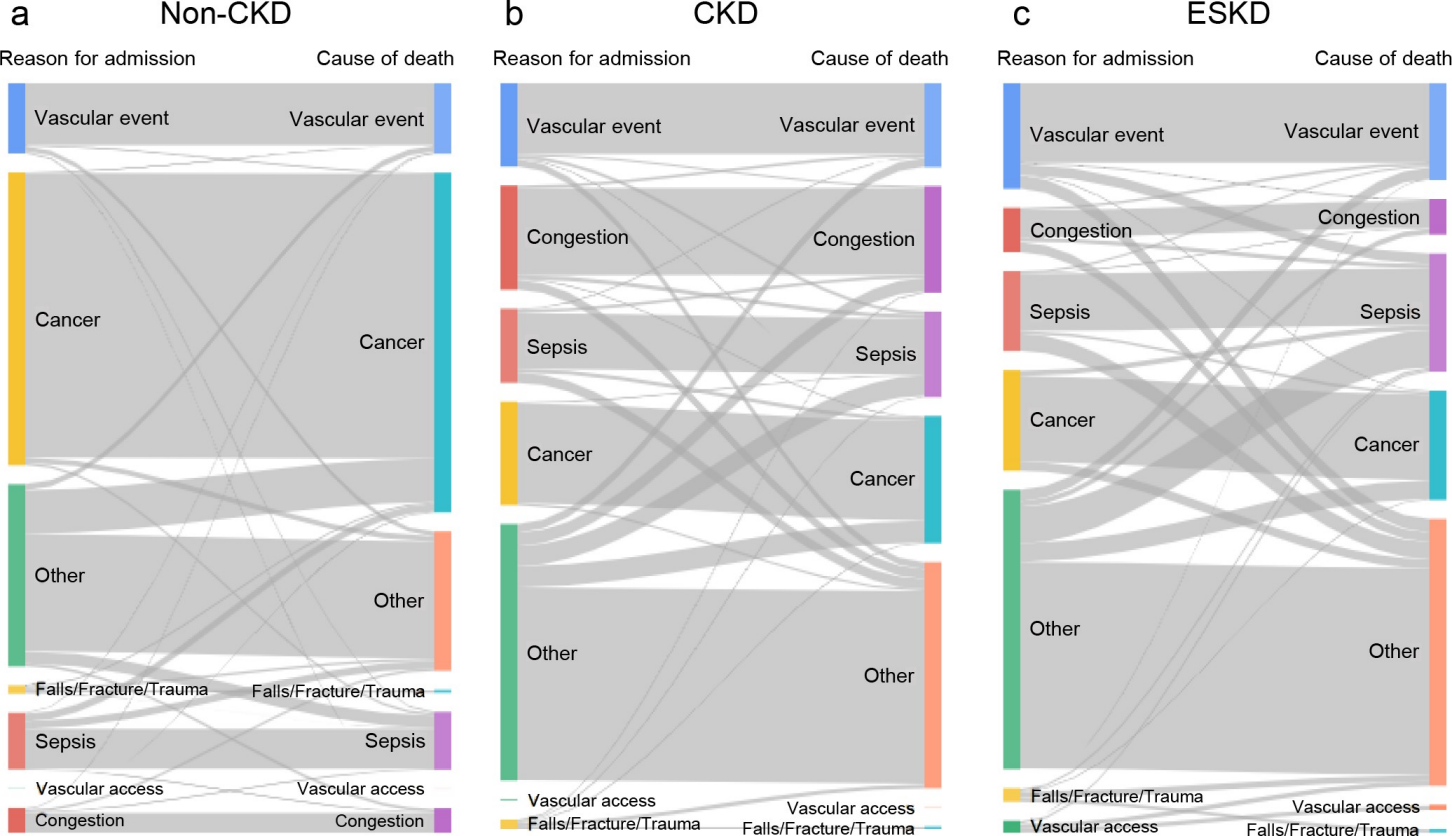
Diagnostic changes in clinical disease classification from hospital admission to in-hospital death among Japanese adults. Seven clinical disease classification categories based on the Healthcare Cost and Utilization Project were applied to Japanese adults with (a) non-CKD, (b) CKD, and (c) ESKD. CKD, chronic kidney disease; ESKD, end-stage kidney disease.

To visually analyze the substantial difference and diagnostic changes in the reasons for hospital admission and death in patients with CKD and ESKD, we illustrated Sankey diagrams representing changes in the seven clinical categories from admission until in-hospital death. As shown in Fig 3, CKD and ESKD patients showed much greater diagnostic changes than the non-CKD population. Most non-CKD adults died of cancer, a finding that accords with previous national administrative data. Among non-CKD patients, few diagnostic changes were seen except for transitions from other disease classifications at the time of hospital admission to cancer as the cause of death, which indicates that a substantial proportion of patients with terminal cancer were initially hospitalized with another problem. Multiple larger changes were evident in CKD and ESKD patients compared to non-CKD patients, particularly the diagnostic change to congestive or septic death, respectively.

To further examine the association between kidney function and in-hospital mortality from specific causes, we performed logistic regression analyses. We also divided the ‘sepsis’ category into ‘pneumonia’ and ‘sepsis excluding pneumonia,’ and 32,144 (5.0%) or 29,541 patients (4.6%) in the whole group were dead due to pneumonia or other sepsis, respectively. As shown in S3 Table in the Supplement, CKD was markedly associated with risk of death from congestion after patients were admitted to the hospital for other reasons. Patients with ESKD had a greater risk of septic death excluding pneumonia after being admitted for other reasons.

### Burden of the discrepancy between reasons for hospital admission and death on length of stay and medical cost

**Fig 4:**
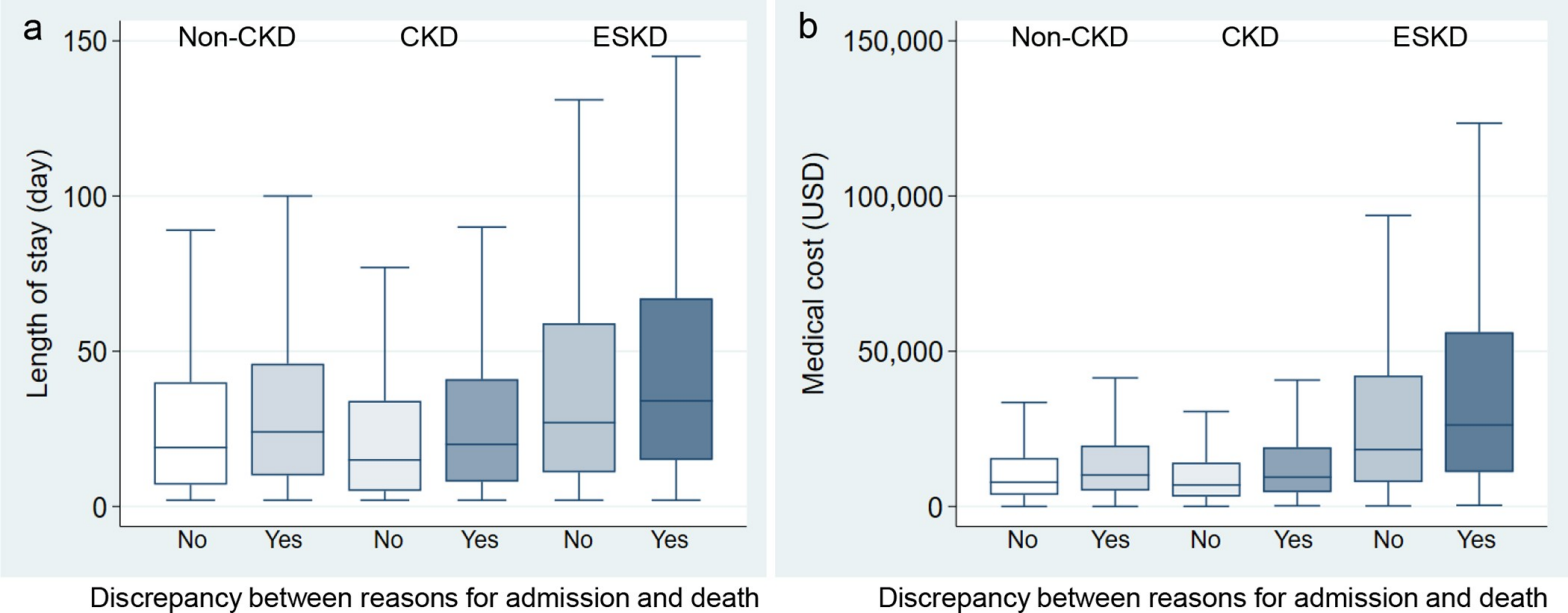
Length of hospital stay and cost among patients who died from the same or distinct disease category than recorded at hospital admission. The graphs show the (a) hospital length of stay and (b) costs that were analysed among Japanese adults who died after hospitalization because of the same or distinct HCUP-based disease classification recorded at hospital admission. Costs were documented using the exchange rate from Japanese yen to United States dollar (USD) on September 9, 2020. CKD, chronic kidney disease; ESKD, end-stage kidney disease; HCUP, Healthcare Cost and Utilization Project; USD, United States dollar.

To investigate the burden of death for unexpected reasons, we examined the length of hospital stay and medical costs for patients with non-CKD, CKD, and ESKD when these patients did or did not die of the same disease classification given at the time of hospital admission. As Fig 4 shows, the median lengths of stay were 19 days (IQR: 7–40), 15 days (IQR: 5–34), and 27 days (IQR: 11–59) for non-CKD, CKD, and ESKD patients, respectively, who died of the reason identified at hospital admission. When the patients died of a different reason after admission, their lengths of stay increased in ≥5 to 24 days (IQR: 10–46), 20 days (IQR: 8–41), and 34 days (IQR: 15–67) for patients with non-CKD, CKD, and ESKD, respectively. As shown in S4 Table, this discrepancy was significantly associated with higher risk for length of stay ≥30 days in the non-CKD, CKD, and ESKD groups, respectively.

The inpatient care costs were 7,787 USD (IQR: 3,712–15,628), 6,906 USD (IQR: 3,168–14,131), and 18,322 USD (IQR: 7,805–42,188) for non-CKD, CKD, and ESKD patients, respectively, without a discrepancy between the reasons for hospital admission and death. In contrast, death from an unexpected reason increased in all populations showing care costs of 10,110 USD (IQR: 5,092–19,618), 9,433 USD (IQR: 4,596–19,044), and 26,239 USD (IQR: 11,078–56,134) for patients with non-CKD, CKD, and ESKD, respectively. Multivariable linear regression analyses showed that the discrepancy was associated with higher costs in all the groups, respectively (S5 Table).

## Discussion

This study clarified the rates, associated factors, and burden of discrepancy between reasons for hospital admission and death in hospitalized adults, which led to longer hospital stays and higher inpatient care expenditures, using a national administrative database. Kidney disease was identified as the leading risk factor for this discrepancy, and 42% of ESKD patients and 30% of CKD patients died during hospitalization of a reason other than the one that they were originally admitted for. Death owing to sepsis or congestion after admission for another problem was a major contributor to the larger discrepancy rates in ESKD and CKD patients, respectively. This study is the first to comprehensively characterize the mismatch and dynamics in clinical diagnoses for hospital admission and death among patients in a nationwide inpatient database.

Clinical experience has indicated that older adults and patients with chronic disease are susceptible to multiple comorbidities and in-hospital death from unexpected reasons. However, prior research lacked objective data. This study showed that one fourth of Japanese adults died of a reason other than the one they were admitted to the hospital for, even when they did not suffer from kidney disease. Surprisingly, over 40% of EKSD patients died from a reason other than that listed at the time of hospital admission. As expected, the associated risk factors for a greater mismatch between reasons for hospital admission and death included older age, male sex, obesity, and comorbidities. Notably, the impact of CKD and particularly ESKD was larger than an increase in single or multiple CCI scores.

ESKD and pre-dialysis CKD are among the major NCDs that affect the functions of multiple organ [1,11–15]. Physicians have been aware that numerous patients with complex diseases die after hospital admission—not because the disease is necessarily the direct cause of death but because a chain of critical comorbidities or events leads to premature death [1–3,24]. Our data showed high proportions of hospital admissions for fractures, trauma, diseases of skin and soft tissue, or vascular access failures leading to in-hospital deaths among CKD and ESKD patients. These findings illustrate the experiences of physicians and explain the higher mortality of CKD and ESKD patients compared to the general population [11].

A greater discrepancy between reasons for hospital admission and death presumably reflects a greater complexity of patients’ problems, difficulties in diagnoses on admission, or risks for newly emerging complications. A recent study of 2.5 million Canadian adults compared the complexity of patients across medical specialties [25]. The patients seen by nephrologists had the highest complexity in terms of the number of comorbidities, number of prescribed medications, rate of death, and rate of placement in long-term care facilities. As patients who visit nephrologists usually suffer from CKD or ESKD, these findings were compatible with our data. We have previously shown that hospital case or dialysis case experience is a key factor to improve ESKD patient outcomes [17]. In particular, a higher HV or DCV was not necessarily associated with a lower discrepancy between the reasons for hospital admission and death (Fig 2). Thus, even if the initial diagnoses are incorrect, prompt diagnoses and initiation of optimal care after admission are essential to decrease patients’ disease complexity, mortality rates, and inpatient care costs.

Notably, the disease category in addition to primary ICD-10 codes greatly changed from hospital admission until death among CKD and ESKD patients. We used Sankey diagrams to visualize multiple wider segments connecting disease classification on hospital admission and death (Fig 3). Death owing to congestion or sepsis after admission with another problem was marked in CKD and EKSD patients, respectively. Earlier correction of volume overload, diagnoses of apparent or unapparent infection, or eradication of the origin of infection are essential to decrease the avoidable deaths of such patients. One explanation for the less discrepancy in more recent years is advancement in inpatient care and diagnostic accuracy on admission. Another explanation might be more repeated admissions due to the overt diagnoses established in outpatient settings, given the upward trends in admission cases.

This study further identified the burden of discrepancy between reasons for hospital admission and death on healthcare expenditures. Death from a reason other than that listed at the time of admission was associated with an increase in the median length of hospital stay by 5–7 days across the groups with various kidney functions and a 1.3-–1.4-fold increase in median cost, which was over 25,000 USD for ESKD adults. The essential reason for this is the need for care of newly occurred complications or multiple comorbidities on admission. The costs for inpatient care at the end of life contribute greatly to the total healthcare expenditures [26,27]. Diagnostic changes during hospitalization might contribute substantially to increasing total healthcare expenditures. Kidney care and CKD-related mortality and costs represent global health challenges, and attempts to decrease avoidable complications during hospitalization and inpatient care costs are essential in healthcare policies.

This study used a large and representative sample of the Japanese population comprising approximately half of the inpatient cases, although the larger hospitals are more likely to participate in the database. A previous study has shown that sensitivity and specificity of primary diagnoses in the DPC database were 78.9% and 93.2%, respectively, supporting the accuracy of the analyses [28]. However, there are several limitations. First, this retrospective study utilized an administrative database with data from a single race and ethnicity; thus, the results may not be generalizable. Second, the cause of death was uncertain in 25% of all the cases. In addition, the study pupation may not include a substantial number of cases in the smaller non-DPC hospitals that play a role in the inter-hospital patient transfer. Third, the number of CKD patients in this study is lower than expected [10]. We additionally analyzed the data under reclassification of the CKD population by adding chronic nephritic syndrome (N030 to N039), nephrotic syndrome (N040 to N049), diabetic kidney disease (N083, E102, E112, and E142) in addition to the N180, N188, and N189 codes. As shown in S3 Fig, the rate of discrepancy between ICD-10 codes on hospital admission and death remained high: 41.6% in ESKD patients (*n* = 585,151), 31.0% in re-classified CKD patients (*n* = 30,108), and 25.6% in re-classified non-CKD patients (*n* = 24,297), similar to the original analysis (Fig 1A). We also revealed higher odds ratios for the discrepancy in the CKD and ESKD groups versus the non-CKD group, respectively (S6 Table). Fourth, this study could not specifically examine if the discrepancy between reasons for admission and death directly increases the risk of eventual death, given the enrolment of patients who died. Further analyses including the intermediate diagnoses that are lacking in this database are needed to clarify this issue.

## Conclusions

Among patients with non-CKD, CKD, and ESKD, 26%, 30%, and 42%, respectively, died during hospitalization of a reason other than that originally recorded at the time of admission. Underlying kidney disease was the predominant risk factor for this diagnostic discrepancy. Death owing to sepsis or congestion after admission for another problem was a major contributor to the larger discrepancy rates in ESKD and CKD patients, respectively. Moreover, death from another reason after hospital admission was associated with an increase in the length of hospital stay and care cost. Attempts to diagnose the primary disease or newly occurred problems earlier and decrease patients’ disease complexity may improve patient outcomes and inpatient care expenditures.

## Supporting information

S1 Fig Patient flowchart. BMI, bone mass index; CKD, chronic kidney disease; ESKD, end-stage kidney disease.
(DOCX)

S2 Fig Rate of in-hospital death due to the undiagnosed diseases at admission among Japanese adults. Each bar graph represents a mean, and the solid lines represent the corresponding 95% confidence interval. CKD, chronic kidney disease; ESKD, end-stage kidney disease; ICD-10, International Classification of Diseases, 10th Revision.
(DOCX)

S3 Fig Rate of discrepancy between primary reasons for hospital admission and death under the reclassification of CKD, ESKD, and non-CKD populations. Rate of discrepancy between primary ICD-10 diagnostic code assigned at the time of hospital admission and death among Japanese adults with non-CKD (*n* = 585,151), CKD (*n* = 30,108), and ESKD (*n* = 24,297). Each bar graph represents a mean, and the solid lines represent the corresponding 95% confidence interval. CKD, chronic kidney disease; ESKD, end-stage kidney disease; ICD-10, International Classification of Diseases, 10th Revision.
(DOCX)

S1 Table Clinical disease classification based on the Healthcare Cost and Utilization Project mapping of ICD-10 diagnosis codes. (DOCX)

S2 Table Factors associated with a discrepancy between disease classification at hospital admission and in-hospital death in logistic regression models among Japanese adults. Seven clinical disease classification categories based on the Healthcare Cost and Utilization Project were applied. Multivariate logistic regression models were adjusted for age, sex, BMI, Charlson comorbidity index, and admission type and year. BMI, body mass index; CI, confidence interval; CKD, chronic kidney disease; ESKD, end-stage kidney disease; OR, odds ratio.
(DOCX)

S3 Table Association of Underlying kidney disease with risk of death from another reason during hospitalization according to various modes of death in logistic regression models among Japanese adults. Clinical disease classification categories based on the Healthcare Cost and Utilization Project were applied. Multivariate logistic regression models were adjusted for age, sex, BMI, Charlson comorbidity index, and admission type and year. BMI, body mass index; CI, confidence interval; CKD, chronic kidney disease; ESKD, end-stage kidney disease; OR, odds ratio.
(DOCX)

S4 Table Association of the discrepancy between primary disease classifications on admission and death with risk of a Long Hospital stay among non-CKD, CKD, and ESKD Japanese adults, respectively. Multivariate logistic regression models were performed to examine the risk of length of stay 30 days, adjusting age, sex, body mass index, Charlson comorbidity index, and admission type and year. Clinical disease classification categories based on the Healthcare Cost and Utilization Project were applied. CI, confidence interval; CKD, chronic kidney disease; ESKD, end-stage kidney disease; OR, odds ratio.
(DOCX)

S5 Table Association of the discrepancy between primary disease classifications on admission and death with costs among non-CKD, CKD, and ESKD Japanese adults, respectively. Multivariate linear regression models were adjusted for age, sex, body mass index, Charlson comorbidity index, and admission type and year. Clinical disease classification categories based on the Healthcare Cost and Utilization Project were applied. Costs were documented using the exchange rate from Japanese yen to United States dollar on September 9, 2020. CI, confidence interval; CKD, chronic kidney disease; ESKD, end-stage kidney disease.
(DOCX)

S6 Table Association of underlying kidney disease with risk of death from another reason during hospitalization under the reclassification of CKD, ESKD, and non-CKD populations. Multivariate logistic regression models were adjusted for age, sex, BMI, Charlson comorbidity index, and admission type and year. BMI, body mass index; CI, confidence interval; CKD, chronic kidney disease; ESKD, end-stage kidney disease; OR, odds ratio.
(DOCX)
