# Supplementary material for: Burden of kidney disease on the discrepancy between reasons for hospital admission and death: An observational cohort study
Source: PLoS One. 2021 Nov 3;16(11):e0258846. doi: 10.1371/journal.pone.0258846 (PMC8565775; doi:10.1371/journal.pone.0258846)
Supplement: S1 Fig — BMI, bone mass index; CKD, chronic kidney disease; ESKD, end-stage kidney disease. (DOCX) [file pone.0258846.s001.docx]

**S1 Fig. Patient flowchart.**


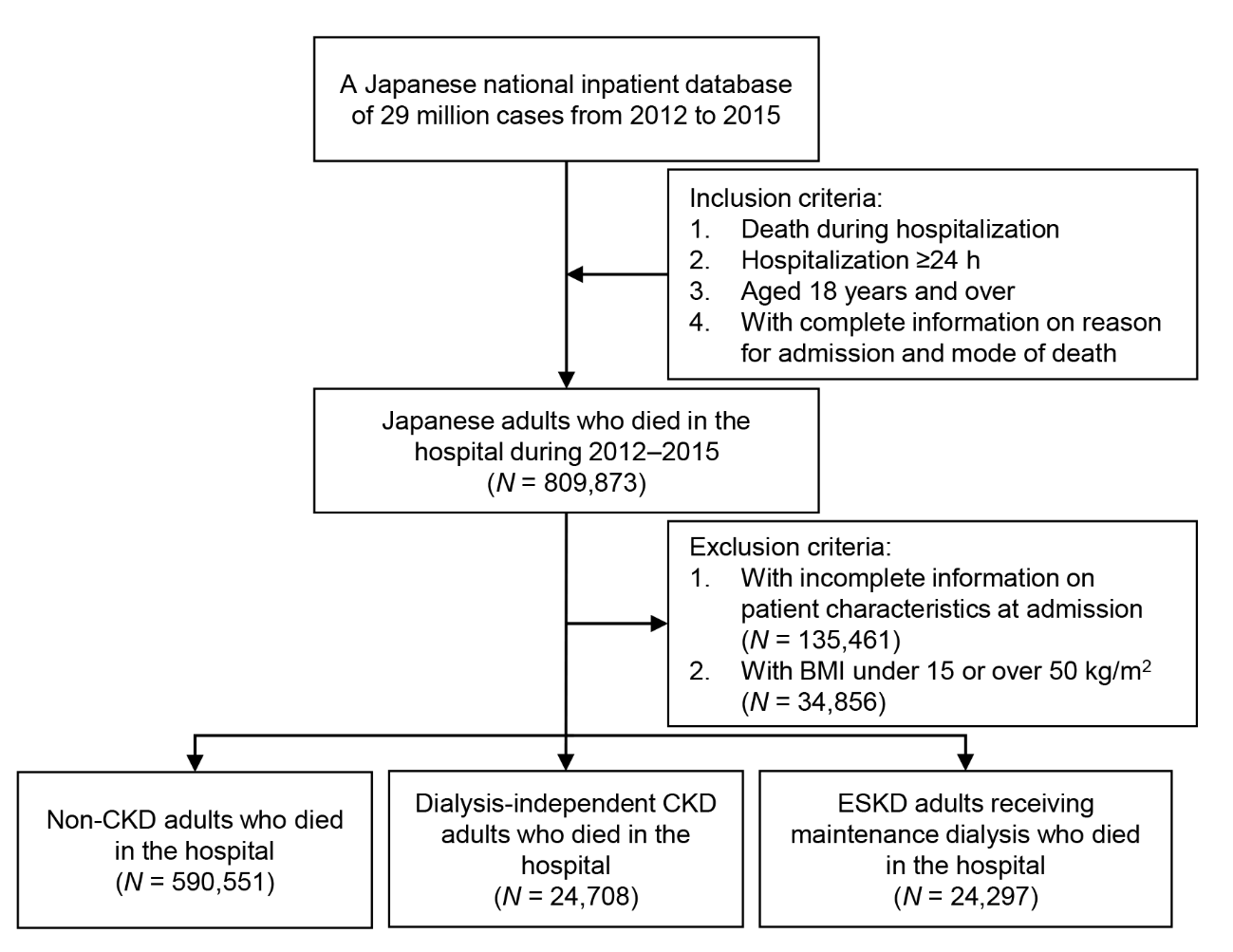


BMI, bone mass index; CKD, chronic kidney disease; ESKD, end-stage kidney disease.
