# Supplementary material for: Burden of kidney disease on the discrepancy between reasons for hospital admission and death: An observational cohort study
Source: PLoS One. 2021 Nov 3;16(11):e0258846. doi: 10.1371/journal.pone.0258846 (PMC8565775; doi:10.1371/journal.pone.0258846)
Supplement: S2 Fig — Each bar graph represents a mean, and the solid lines represent the corresponding 95% confidence interval. CKD, chronic kidney disease; ESKD, end-stage kidney disease; ICD-10, International Classification of Diseases, 10th Revision. (DOCX) [file pone.0258846.s002.docx]

**S2 Fig. Rate of in-hospital death due to the undiagnosed diseases at admission among Japanese adults.**

**
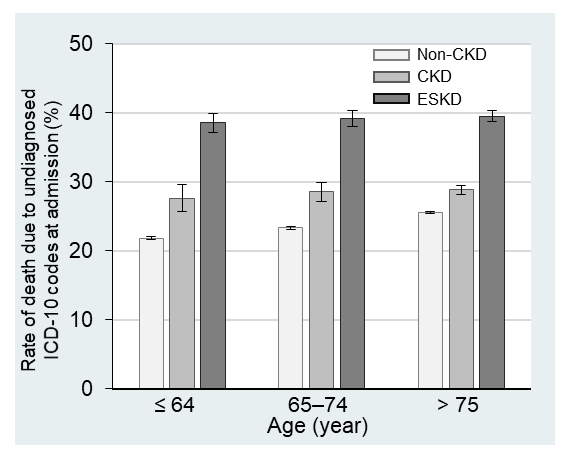
**

Each bar graph represents a mean, and the solid lines represent the corresponding 95% confidence interval. CKD, chronic kidney disease; ESKD, end-stage kidney disease; ICD-10, International Classification of Diseases, 10th Revision.
