# Supplementary material for: Burden of kidney disease on the discrepancy between reasons for hospital admission and death: An observational cohort study
Source: PLoS One. 2021 Nov 3;16(11):e0258846. doi: 10.1371/journal.pone.0258846 (PMC8565775; doi:10.1371/journal.pone.0258846)
Supplement: S3 Fig — Rate of discrepancy between primary ICD-10 diagnostic code assigned at the time of hospital admission and death among Japanese adults with non-CKD (n = 585,151), CKD (n = 30,108), and ESKD (n = 24,297). Each bar graph represents a mean, and the solid lines represent the corresponding 95% confidence interval. CKD, chronic kidney disease; ESKD, end-stage kidney disease; ICD-10, International Classification of Diseases, 10th Revision. (DOCX) [file pone.0258846.s003.docx]

**S3 Fig. Rate of discrepancy between primary reasons for hospital admission and death under the reclassification of CKD, ESKD, and non-CKD populations.**

**
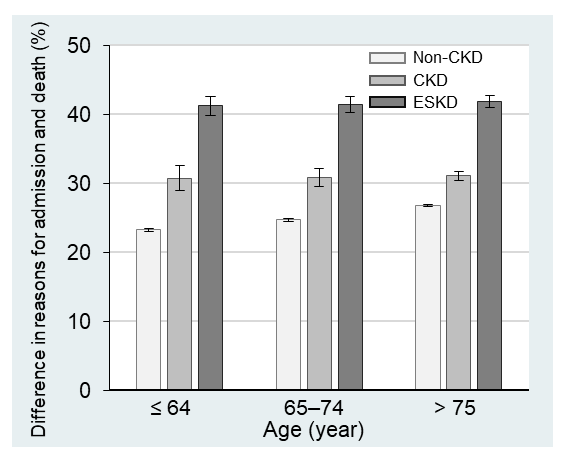
**

Rate of discrepancy between primary ICD-10 diagnostic code assigned at the time of hospital admission and death among Japanese adults with non-CKD (*n* = 585,151), CKD (*n* = 30,108), and ESKD (*n* = 24,297). Each bar graph represents a mean, and the solid lines represent the corresponding 95% confidence interval. CKD, chronic kidney disease; ESKD, end-stage kidney disease; ICD-10, International Classification of Diseases, 10th Revision.
