# Supplementary material for: Burden of kidney disease on the discrepancy between reasons for hospital admission and death: An observational cohort study
Source: PLoS One. 2021 Nov 3;16(11):e0258846. doi: 10.1371/journal.pone.0258846 (PMC8565775; doi:10.1371/journal.pone.0258846)
Supplement: S1 Table — (DOCX) [file pone.0258846.s004.docx]

**S1 Table. Clinical disease classification based on the Healthcare Cost and Utilization Project mapping of ICD-10 diagnosis codes.**

| **Disease category** | **HCUP Clinical Classification Software Group(s)** |
| --- | --- |
| 1. Vascular Event | Coronary atherosclerosis (101), Other circulatory disease (117), Acute myocardial infarction (100), Peripheral and visceral atherosclerosis (114), Chronic ulcer of skin (199), Gangrene (248), Aortic; peripheral; and visceral arterial disease (115), Aortic and peripheral arterial emboli (116), Transient cerebral ischemia (112), Cardiac arrest and ventricular fibrillation (107), Pulmonary heart disease (103), Other and ill-defined cerebrovascular disease (111), Acute cerebrovascular disease (109) |
| 2. Congestion | Congestive heart failure; nonhypertensive (108) |
| 3. Sepsis | Pneumonia (122), Septicemia (except in labor) (2), Pleurisy; pneumothorax; pulmonary collapse (130), Tuberculosis (1), Mycoses(4), HIV infection(5), Encephalitis (77), Meningitis (76), Skin and subcutaneous tissue infection (197), Infective arthritis and osteomyelitis(201), Bacterial infection; unspecified site (3), Other infections; including parasitic (8), Influenza (123), Urinary tract infections (159) |
| 4. Cancer | Cancer of head and neck (11), Cancer of esophagus (12), Cancer of stomach (13), Cancer of colon (14), Cancer of rectum and anus (15), Cancer of liver and intrahepatic bile duct (16), Cancer of pancreas (17), Cancer of other GI organs; peritoneum (18), Cancer of bronchus; lung (19), Cancer; other respiratory and intrathoracic (20), Cancer of bone and connective tissue (21), Melanomas of skin (22), Other non-epithelial cancer of skin (23), Cancer of breast (24), Cancer of uterus (25), Cancer of cervix (26), Cancer of ovary (27), Cancer of other female genital organs (28), Cancer of prostate (29), Cancer of testis (30), Cancer of other male genital organs (31), Cancer of bladder (32), Cancer of kidney and renal pelvis (33), Cancer of other urinary organs (34), Cancer of brain and nervous system (35), Cancer of thyroid (36), Hodgkin’s disease (37), Non-Hodgkin’s lymphoma (38), Leukemias (39), Multiple myeloma (40), Cancer; other and unspecified primary (41), Secondary malignancies (42), Malignant neoplasm without specification of site (43), Neoplasms of unspecified nature or uncertain behavior (44) |
| 5. Falls/Fracture/Trauma | Fracture of neck of femur (hip) (226), Superficial injury; contusion (239), Fracture of lower limb (230), Other fractures (231), Fracture of upper limb (229), Crushing injury or internal injury (234), Open wounds of head; neck; and trunk (235), Pathological fracture (207), Open wounds of extremities (236), Other injuries and conditions due to external causes (244), Joint disorders and dislocations; trauma related (225), Sprains and strains (232), Skull and face fractures (228) |
| 6. Vascular Access | Complication of device, implant or graft (237) |
| 7. Other | Remaining codes |
