# Supplementary material for: Burden of kidney disease on the discrepancy between reasons for hospital admission and death: An observational cohort study
Source: PLoS One. 2021 Nov 3;16(11):e0258846. doi: 10.1371/journal.pone.0258846 (PMC8565775; doi:10.1371/journal.pone.0258846)
Supplement: S2 Table — Seven clinical disease classification categories based on the Healthcare Cost and Utilization Project were applied. Multivariate logistic regression models were adjusted for age, sex, BMI, Charlson comorbidity index, and admission type and year. BMI, body mass index; CI, confidence interval; CKD, chronic kidney disease; ESKD, end-stage kidney disease; OR, odds ratio. (DOCX) [file pone.0258846.s005.docx]

**S2 Table. Factors associated with a discrepancy between disease classification at hospital admission and in-hospital death in logistic regression models among Japanese adults.**

| **Variable** | **OR (95%CI)** | ***P* value** |
| --- | --- | --- |
| Kidney disease |  |  |
| Non-CKD | Reference |  |
| CKD | 1.128 (1.092 to 1.165) | <0.001 |
| ESKD | 1.904 (1.848 to 1.961) | <0.001 |
| Age (year) |  |  |
| ≤ 64 | Reference |  |
| 65–74 | 1.131 (1.107 to 1.155) | < 0.001 |
| ≥ 75 | 1.217 (1.195 to 1.239) | < 0.001 |
| Sex |  |  |
| Female vs. male | 0.973 (0.960 to 0.986) | < 0.001 |
| BMI (kg per m^2^) |  |  |
| ≤ 18 | 1.001 (0.986 to 1.015) | 0.9 |
| 19–24 | Reference |  |
| 25–29 | 1.004 (0.984 to 1.025) | 0.4 |
| ≥ 30 | 1.068 (1.025 to 1.113) | < 0.001 |
| Charlson comorbidity index | 1.030 (1.028 to 1.033) | < 0.001 |
| Admission type |  |  |
| Emergent vs. elective | 2.025 (1.985 to 2.065) | <0.001 |
| Year |  |  |
| 2012 | Reference |  |
| 2013 | 0.965 (0.947 to 0.983) | <0.001 |
| 2014 | 0.923 (0.907 to 0.940) | <0.001 |
| 2015 | 0.854 (0.838 to 0.870) | <0.001 |

Seven clinical disease classification categories based on the Healthcare Cost and Utilization Project were applied. Multivariate logistic regression models were adjusted for age, sex, BMI, Charlson comorbidity index, and admission type and year. BMI, body mass index; CI, confidence interval; CKD, chronic kidney disease; ESKD, end-stage kidney disease; OR, odds ratio.
