# Supplementary material for: Burden of kidney disease on the discrepancy between reasons for hospital admission and death: An observational cohort study
Source: PLoS One. 2021 Nov 3;16(11):e0258846. doi: 10.1371/journal.pone.0258846 (PMC8565775; doi:10.1371/journal.pone.0258846)
Supplement: S3 Table — Clinical disease classification categories based on the Healthcare Cost and Utilization Project were applied. Multivariate logistic regression models were adjusted for age, sex, BMI, Charlson comorbidity index, and admission type and year. BMI, body mass index; CI, confidence interval; CKD, chronic kidney disease; ESKD, end-stage kidney disease; OR, odds ratio. (DOCX) [file pone.0258846.s006.docx]

**S3 Table.** **Association of underlying kidney disease with risk of death from another reason during Hospitalization according to various modes of death in logistic regression models among Japanese adults.**

| **Variable** | **OR (95%CI)** | ***P* value** |
| --- | --- | --- |
| Death from all causes |  |  |
| Non-CKD | Reference |  |
| CKD | 1.128 (1.092 to 1.165) | <0.001 |
| ESKD | 1.904 (1.848 to 1.961) | <0.001 |
| Death from vascular event |  |  |
| Non-CKD | Reference |  |
| CKD | 1.309 (1.193 to 1.429) | <0.001 |
| ESKD | 1.966 (1.814 to 2.131) | <0.001 |
| Death from congestion |  |  |
| Non-CKD | Reference |  |
| CKD | 3.143 (2.905 to 3.400) | <0.001 |
| ESKD | 1.859 (1.660 to 2.082) | <0.001 |
| Death from pneumonia |  |  |
| Non-CKD | Reference |  |
| CKD | 1.167 (1.055 to 1.291) | 0.003 |
| ESKD | 1.010 (0.895 to 1.140) | 0.9 |
| Death from sepsis excluding pneumonia |  |  |
| Non-CKD | Reference |  |
| CKD | 1.518 (1.414 to 1.630) | <0.001 |
| ESKD | 4.530 (4.318 to 4.752) | <0.001 |
| Death from cancer |  |  |
| Non-CKD | Reference |  |
| CKD | 0.545 (0.512 to 0.579) | <0.001 |
| ESKD | 0.481 (0.450 to 0.515) | <0.001 |
| Death from other disease |  |  |
| Non-CKD | Reference |  |
| CKD | 1.378 (1.300 to 1.460) | <0.001 |
| ESKD | 2.784 (2.660 to 2.914) | <0.001 |

Seven clinical disease classification categories based on the Healthcare Cost and Utilization Project were applied. Multivariate logistic regression models were adjusted for age, sex, BMI, Charlson comorbidity index, and admission type and year. BMI, body mass index; CI, confidence interval; CKD, chronic kidney disease; ESKD, end-stage kidney disease; OR, odds ratio.
