# Supplementary material for: Burden of kidney disease on the discrepancy between reasons for hospital admission and death: An observational cohort study
Source: PLoS One. 2021 Nov 3;16(11):e0258846. doi: 10.1371/journal.pone.0258846 (PMC8565775; doi:10.1371/journal.pone.0258846)
Supplement: S4 Table — Multivariate logistic regression models were performed to examine the risk of length of stay 30 days, adjusting age, sex, body mass index, Charlson comorbidity index, and admission type and year. Clinical disease classification categories based on the Healthcare Cost and Utilization Project were applied. CI, confidence interval; CKD, chronic kidney disease; ESKD, end-stage kidney disease; OR, odds ratio. (DOCX) [file pone.0258846.s007.docx]

**S4 Table. Association of the discrepancy between primary disease classifications on admission and death with risk of a long hospital stay among Non-CKD, CKD, and ESKD Japanese adults, respectively.**

|  | **Univariate** | |  | **Multivariable** | |
| --- | --- | --- | --- | --- | --- |
| **Variable** | **OR (95%CI)** | ***P* value** |  | **OR (95%CI)** | ***P* value** |
| Non-CKD |  |  |  |  |  |
| No discrepancy | Reference |  |  | Reference |  |
| Discrepancy | 1.33 (1.31 to 1.35) | <0.001 |  | 1.46 (1.44 to 1.48) | <0.001 |
| CKD |  |  |  |  |  |
| No discrepancy | Reference |  |  | Reference |  |
| Discrepancy | 1.34 (1.25 to 1.43) | <0.001 |  | 1.37 (1.28 to 1.47) | <0.001 |
| ESKD |  |  |  |  |  |
| No discrepancy | Reference |  |  | Reference |  |
| Discrepancy | 1.35 (1.28 to 1.43) | <0.001 |  | 1.37 (1.30 to 1.46) | <0.001 |

Multivariable logistic regression models were performed to examine the risk of length of stay 30 days, adjusting age, sex, body mass index, Charlson comorbidity index, and admission type and year. Clinical disease classification categories based on the Healthcare Cost and Utilization Project were applied. CI, confidence interval; CKD, chronic kidney disease; ESKD, end-stage kidney disease; OR, odds ratio.
