# Supplementary material for: Burden of kidney disease on the discrepancy between reasons for hospital admission and death: An observational cohort study
Source: PLoS One. 2021 Nov 3;16(11):e0258846. doi: 10.1371/journal.pone.0258846 (PMC8565775; doi:10.1371/journal.pone.0258846)
Supplement: S5 Table — Multivariate linear regression models were adjusted for age, sex, body mass index, Charlson comorbidity index, and admission type and year. Clinical disease classification categories based on the Healthcare Cost and Utilization Project were applied. Costs were documented using the exchange rate from Japanese yen to United States dollar on September 9, 2020. CI, confidence interval; CKD, chronic kidney disease; ESKD, end-stage kidney disease. (DOCX) [file pone.0258846.s008.docx]

**S5 Table. Association of the discrepancy between primary disease classifications on admission and death with costs among non-CKD, CKD, and ESKD Japanese adults, respectively.**

|  | **Univariate** | |  | **Multivariable** | |
| --- | --- | --- | --- | --- | --- |
| **Variable** | **Coefficient (95%CI)** | ***P* value** |  | **Coefficient (95%CI)** | ***P* value** |
| Non-CKD |  |  |  |  |  |
| No discrepancy | Reference |  |  | Reference |  |
| Discrepancy | 3,169 (3,001 to 3,337) | <0.001 |  | 4,351 (4,186 to 4,516) | <0.001 |
| CKD |  |  |  |  |  |
| No discrepancy | Reference |  |  | Reference |  |
| Discrepancy | 4,120 (3,439 to 4,800) | <0.001 |  | 4,496 (3,826 to 5,166) | <0.001 |
| ESKD |  |  |  |  |  |
| No discrepancy | Reference |  |  | Reference |  |
| Discrepancy | 5,935 (4,305 to 7,565) | <0.001 |  | 6,923 (5,363 to 8,483) | <0.001 |

Multivariable linear regression models were adjusted for age, sex, body mass index, Charlson comorbidity index, and admission type and year. Clinical disease classification categories based on the Healthcare Cost and Utilization Project were applied. Costs were documented using the exchange rate from Japanese yen to United States dollar on September 9, 2020. CI, confidence interval; CKD, chronic kidney disease; ESKD, end-stage kidney disease.
