# Supplementary material for: Burden of kidney disease on the discrepancy between reasons for hospital admission and death: An observational cohort study
Source: PLoS One. 2021 Nov 3;16(11):e0258846. doi: 10.1371/journal.pone.0258846 (PMC8565775; doi:10.1371/journal.pone.0258846)
Supplement: S6 Table — Multivariate logistic regression models were adjusted for age, sex, BMI, Charlson comorbidity index, and admission type and year. BMI, body mass index; CI, confidence interval; CKD, chronic kidney disease; ESKD, end-stage kidney disease; OR, odds ratio. (DOCX) [file pone.0258846.s009.docx]

**S6 Table. Association of underlying kidney disease with risk of death from another reason during hospitalization under the reclassification of CKD, ESKD, and non-CKD populations.**

| **Variable** | **OR (95%CI)** | ***P* value** |
| --- | --- | --- |
| Non-CKD | Reference |  |
| CKD | 1.268 (1.237 to 1.301) | <0.001 |
| ESKD | 2.232 (2.174 to 2.292) | <0.001 |

Multivariate logistic regression models were adjusted for age, sex, BMI, Charlson comorbidity index, and admission type and year. BMI, body mass index; CI, confidence interval; CKD, chronic kidney disease; ESKD, end-stage kidney disease; OR, odds ratio.
